# The Optimal Cut-Off Point of Physical Activity for the Prevention of Childhood Overweight and Obesity

**DOI:** 10.3390/children11050569

**Published:** 2024-05-09

**Authors:** Shuxian Wu, Yu Huang, Lei Wang, Xiang Zhao, Qiaohong Lv, Qingqing Wu

**Affiliations:** Zhejiang Provincial Center for Disease Control and Prevention, Hangzhou 310051, China; sxwu@cdc.zj.cn (S.W.); yhuang@cdc.zj.cn (Y.H.); leiwang@cdc.zj.cn (L.W.); xzhao@cdc.zj.cn (X.Z.); qhlv@cdc.zj.cn (Q.L.)

**Keywords:** physical activity, overweight, obesity, children, adolescents

## Abstract

Childhood obesity might threaten children’s current and adulthood health outcomes. Previous studies have illustrated the positive effects of physical activity on weight control; however, there is a lack of evidence on the optimal dose of physical activity. Therefore, we aimed to explore the relationship between physical activity and overweight and obesity, as well as the optimal threshold for physical activity. The median (interquartile range) and number (proportion) were used to describe the statistics. The Mann–Whitney U test and chi-square test were used for an univariable analysis. The generalized additive model with a smooth function was used to depict the preliminary relationship between physical activity and overweight and obesity. The cut-off level of physical activity was identified using AddFor algorithms, and a logistic regression model was applied to explore the multivariable relationship between physical activity and overweight and obesity after adjusting for control variables. According to the statistical analyses, 24.3% of 842 children and adolescents had overweight and obesity. The average number of days that the participants engaged in more than one hour of physical activity was three days a week. The optimal cut-off level of physical activity for the prevention of childhood overweight and obesity was 4 times a week. The participants who exercised more than four times a week (A*OR*(95% *CI*) = 0.56(0.38, 0.83), *p* = 0.004) were less likely to be overweight and obese. In the context of the general lack of physical activity among children and adolescents, we recommend that children and adolescents engage in 60 min of physical activity more than four times a week for a healthy weight.

## 1. Introduction

Overweight and obesity are important health issues among children and adolescents aged 5–17 years. More than 340 million children are at risk of being overweight or obese, accounting for more than 18% of the adolescent population worldwide, and the prevalence is still on the rise [1,2]. The prevalence of overweight and obesity among Chinese children and adolescents has been increasing year by year, from “basically non-existent” in the 1980s to a “rapid increase” in the 1990s and to “rising continuously” in the 21st century. Currently, about 19% of Chinese children and adolescents are overweight or obese [3]. Overweight and obesity that begin in childhood and adolescence might persist throughout life and lead to childhood integration problems, a lack of self-efficacy, and non-communicable diseases in adulthood. Studies have revealed that in 50% of children with obesity and in 80% of youth with severe obesity, adulthood severe obesity might occur. Obese children and adolescents have more than twice the risk of dyslipidemia, hypertension, type 2 diabetes, and metabolic diseases than non-obese groups. Childhood obesity is associated with a more than twofold increased odds of kidney-related diseases. The higher the BMI in childhood, the higher the risk of corresponding diseases in adulthood [4,5,6,7].

According to the bio-socioecological framework, multiple factors account for childhood obesity, ranging from the individual, family peers, childcare and school, community and built environment, and society to the public policy level [2]. However, weight fitness management cannot be separated from energy intake and energy expenditure.

Studies have shown that adequate and regular physical activity reduces the risk of overweight and obesity among adults, and, in those who are already obese, physical activity also improves insulin action, blood lipids, endothelial function, and blood pressure, thus reducing comorbidities [8]. Similarly, adequate physical activity can also reduce the risk of overweight and obesity in children and adolescents by 7% [9]. Numerous studies have found positive impacts of physical activity on preventing pediatric obesity [2,9,10,11]. The recommendation for physical activity for children and adolescents is much higher than that for adults. The WHO recommends 150–300 min of physical activity for adults per week but an average of at least 60 min per day of moderate-to-vigorous intensity physical activity for children and adolescents aged 5–17 years for fitness [12]. However, the optimal physical activity dose associated with weight has not been determined because of limited available data.

More than 80% of children and adolescents do not meet the WHO recommended levels of physical activity, falling far short of the requirements of the Global Action Plan on Physical Activity 2018–2030 [13]. A lack of physical activity among children and adolescents results from many factors, ranging from personal willingness, interpersonal influences, facilities, and equipment environments to sociocultural environments. Overall, costs and time are barriers to increasing physical activity [14,15]. For children, leisure time apart from schoolwork is very limited. Research has shown that children and adolescents have no more than two hours of leisure time per day. Furthermore, in the past decade, children and adolescents have started to spend more time on-screen viewing. Screen exposure time has risen by 1.16 times. School-aged children who use social media more frequently are less likely to participate in physical activity. At the same time, with the increase in grades and academic burden, children and adolescents have less time to spend on physical activity and other leisure activities [16]. Therefore, it is crucial for school-aged children and adolescents to strike a balance between school work and physical activity. Identifying the optimal cut-off point is helpful to refine the recommended dose and further understand the relationship between physical activity and childhood overweight and obesity.

Generalized additive models have some advantages in exploring the relationship between variables. It is a general class of models that allow for parametric and nonparametric forms of relationship between variables. A smoothing spline is used for fitting wiggly curves. Generally, they make no assumption of linearity between predictors and outcome variables and can depict complex, non-linear relationships between variables [17,18]. The area under the receiver operating characteristic curve is a popular measure of pure diagnostic accuracy, and it is used to assess the accuracy of cut-off points.

The present study is a secondary analysis of the Chinese Youth Health Study, which aimed to explore the relationship between physical activity and overweight and obesity among children and adolescents in Zhejiang Province and then determine the optimum threshold for physical activity associated with childhood overweight and obesity. Based on this aim, we hypothesized that physical activity time that exceeded the threshold would be correlated with a lower BMI and overweight and obesity risks. Control variables such as family economic level, gender, and grades were included in our analysis to address the potential confounders linked to childhood overweight and obesity. This study demonstrates an evidence-based dose–response relationship between physical activity and childhood overweight and obesity. In addition, it can be used to guide children and adolescents to engage in adequate physical activity in a limited time to prevent childhood overweight and obesity.

## 2. Methods

### 2.1. Participants

Participants were selected from the Zhejiang-Chinese Youth Health Study [19]. The Chinese Youth Health Study was a school-based cross-sectional study originating from the WHO-School Age Children’s Health Behavior Project, and it aimed to explore the relationships between children’s and adolescents’ behaviors and health outcomes. Considering socioeconomic level, geographical location, and enrolment in a public or private school, an online questionnaire survey was conducted from September to November 2020 in 13 primary and secondary schools in Zhejiang Province. The 13 schools were located in cities with different economic levels in Zhejiang Province and consisted of both public and private schools. The cluster random sampling method was used. All students in one grade 5 class in primary school, one grade 2 class in junior high school, and one grade 1 class and one grade 2 class in senior high school were invited to participate in the survey. Children and adolescents aged 11, 13, 15, and 17 years old were selected in order to understand the development of and changes in health-related behaviors and outcomes at early, middle, and late puberty [19,20]. In China, these ages correspond to students in the abovementioned survey grades. Therefore, we conducted surveys of this population.

In this analysis, participants who were in the above-selected classes were included. Additionally, participants (1) who had dyslexia or were unable to complete the survey for other reasons and (2) who did not report their height and weight or whose weight and height were more than 3 times the interquartile range were excluded. Finally, a total of 842 children and adolescents were included in the statistical analysis. All participants were informed of the aim of the research and signed informed consent forms.

### 2.2. Measurements

#### 2.2.1. Predictive Variables

Physical activity time was measured by asking the participants how many days they engaged in more than one hour of physical exercise in the previous week [13,21].

#### 2.2.2. Outcome Variable

The participant’s weight and height were collected from a self-report questionnaire, and their BMI was calculated from the questionnaire results. Overweight and obesity among children and adolescents were determined via sex- and age-specific BMI, according to the national screening standard for overweight and obesity among school-age children and adolescents [22]. The participants were divided into two groups (a non-overweight group or an overweight and obesity group).

#### 2.2.3. Control Variables

Based on the socioecological model for child and adolescent obesity and a literature review, the control variables in this study included factors from the individual and family levels [2]. Family-level variables included family socioeconomic position and screen behavior [2]. The Family Affluence Scale (FAS) was used to measure family socioeconomic level [23]. This scale consists of four items, which are used to calculate the number of cars, computers, private bedrooms, and trips in a family. The total score of the FAS is 9 points, which is divided into three subgroups (low, medium, and high family economic level) [23]. Concerning the platforms and purpose of childhood screen exposure, the total time spent on computers, tablets, mobile phones, and other electronic devices in a week for (a) online studying and doing homework, (b) watching videos, (c) sending emails and chatting, and (d) playing games were added as screen exposure duration per week [24]. Individual-level variables included sex (male or female), grades (primary, junior high, or senior high school student), household registration status (urban or rural household), and left-behind situation (non or left-behind children) [2].

### 2.3. Data Analyses

First, in view of the non-normality distribution of the variables, the median (interquartile range, *IDQ*) and number (proportion) were used to describe the statistics for continuous and count variables. The Mann–Whitney U test was used for continuous variables (screen exposure duration) and the chi-square test was used for categorical variables (sex, grades, household registration status, left-behind situation, and family economic level) to explore the relationships between the non-overweight and overweight and obesity groups. Second, to explore potential preliminarily nonlinear relationships among physical activity time and overweight and obesity, generalized additive models (GAMs) with a logit link function were utilized after adjusting for control variables. The number of cut-off points was determined by observing the shape of the curve. Third, the optimal cut-off points for the physical activity time variable were identified using AddFor algorithms. AddFor algorithms categorize continuous predictors within a regression model in such a way that the best discriminative ability is obtained in terms of the maximized *AUC* (area under the receiver operating characteristic curve). The advantages of this algorithm are that, first, control variables can be included and that, second, the number of cut-off points can be set by researchers [17,25,26]. A multivariable analysis was used to explore the relationship between categorized predictive variables and outcome variables after adjusting for control variables. Adjusted odds ratios (A*OR*s) and 95% *CI*s for regression models were calculated to describe the association between each independent variable. The statistically significant alpha level was set to 0.05. Statistical analyses were performed using R software, version 4.3.1.

## 3. Results

(a) General sociodemographic information of 842 participants

Of the 842 participants, 464 (55.1%) were female. A total of 205 children and adolescents were determined to have overweight and obesity, accounting for 24.3% of the total population. A total of 177 (21.0%) primary school students, 183 (21.6%) junior high school students, and 483 (57.4%) high school students were surveyed. The Mann–Whitney U test showed that the proportion of participants who were overweight and obese increased with rising grades (*p* < 0.001), from 177 (21%) in primary school to 182 (21.6%) in junior high school and 483 (57.4%) in senior high school. More than half of the participants (444, 52.7%) had urban household registration. Most of them (781, 92.8%) were non-left-behind children. The proportion of participants with a low, middle, and high family economic level was 19.8%, 55.7%, and 24.5%, respectively. The average number of days that the children and adolescents engaged in more than one hour of physical activity was three days a week. The average number of days engaged in physical activity that induced panting and sweating was 4 (*IDQ* = 4) days a month. The participants spent 21 (*IDQ* = 11) hours a week on average on-screen viewing. The relationships between sex, household registration status, left-behind situation, family economic level, and participants’ weight showed no statistical significance (Table 1).

(b) Relationship between physical activity time and overweight and obesity using GAM model

**Figure 1:**
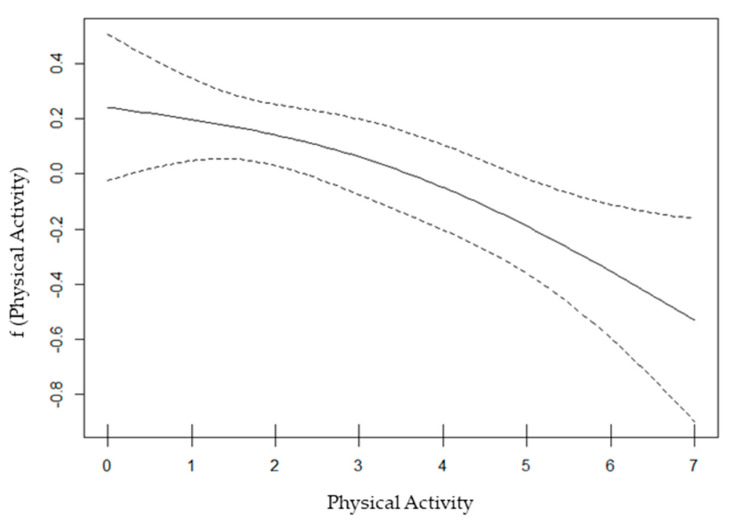
Curvilinear relationship between physical activity time and prevalence. Dashed lines: 95% Confidence Limits.

The results show that the nonlinear relationship between physical activity time and overweight and obesity was statistically significant (*edf* = 1.52, *p* = 0.016). When the value of *edf* was closer to 1, the relationship between the two variables tended to be linear. In Figure 1, the horizontal axis represents the days of the week with more than one hour of physical activity, and the vertical axis reflects the smoothed logit form of the overweight and obesity prevalence. Figure 1 shows an overall decreasing trend in the curvilinear relationship between physical activity time and overweight and obesity prevalence with increasing slopes. The increasing slopes indicate that the effect of physical activity on overweight and obesity became greater as the physical activity time increased. Despite the differences in the slopes, the overall trend of the curve is downward, which illustrates that, with increasing physical activity time, children and adolescents were less likely to be overweight and obese. Based on the overall consistent direction of the curve and without reverse points, we set the number of cut-off points to 1.

(c) Results of multivariable analysis

An analysis showed that, to obtain a dichotomous variable for the outcomes, the threshold for the physical activity time variable should be set to 4 days a week (*AUC* = 0.64, *p* < 0.001). The multivariable analysis revealed that, after controlling for sex, grades, household registration status, left-behind situation, family economic level, and screen exposure duration, the children and adolescents who engaged in more than one hour of physical activity four days a week were less likely to have overweight and obesity (A*OR* = 0.56, 95% *CI* = 0.38, 0.83, *p* = 0.004). The risk of overweight and obesity in senior students was significantly higher than in junior and primary students (junior high school students: A*OR* = 0.53, 95% *CI* = 0.33, 0.82, *p* = 0.006; senior high school students: A*OR* = 0.34, 95% *CI* = 0.22, 0.51, *p* < 0.001, primary school students: reference group). The multivariable analysis showed no statistical significance between sex, household registration status, left-behind situation, family economic level, and screen exposure duration between the participants with overweight and obesity (Table 2).

## 4. Discussion

This study aimed to identify the optimal threshold for physical activity in order to prevent childhood overweight and obesity. Four times a week was identified to be the optimal cut-off, underlining the positive effect of adequate physical activity on healthy weight among children and adolescents.

Overweight and obesity are complex in children. In addition, pediatric overweight and related peri-pubertal behavior patterns will persist throughout their lifetime [27]. According to the bio-socioecological framework, the increase in the prevalence of obesity is caused by factors ranging from the individual to the public policy level [2]. Our research revealed that the prevalence rate of overweight and obesity increased with grades and peaked in high school. A relationship between grades and overweight and obesity has been found in several studies. The cause of this trend might be due to more sedentary behavior and less physical activity [28]. Studies have indicated that a decrease in physical activity time is widespread among upper-year students. Students in high grades spend about one-third as much time in physical activity as younger students [13,29].

The relationship between screen exposure and overweight remains controversial. This is partly because previous studies did not distinguish screen time in terms of sedentary and active video time [30]. In the 1990s, screen exposure in children mainly consisted of watching TV programs; however, children and adolescents now usually use mobile smart devices to surf the internet. Equipment, form, and contents of screen exposure have changed considerably. Previous studies focused on exploring the relationship between traditional media and overweight, and they pointed out that children usually eat while viewing, thus increasing the risk of obesity [24]. However, because of the lockdown during the COVID-19 pandemic, indoor activities increased substantially [31]. Recent studies have shown that active video games might increase physical activity time and are good for preventing childhood overweight and obesity [32,33,34]. In our study, the statistical association between screen exposure and overweight disappeared in the multivariable analyses when physical activity time was included. This suggests that, in the era of mobile media, screen exposure may contribute to overweight or obesity by affecting physical activity levels in children and adolescents.

Physical activity provides many health benefits in both the physical and mental health domains, and it helps to improve interpersonal relationships [13]. Although a few studies have suggested that physical activity increases the proportion of lean muscle mass or excessive energy consumption and then increases BMI [35], mainstream studies still advocate for more physical activity against the backdrop of a general lack of physical activity among children and adolescents. The physical activity level of children and adolescents in our study did not meet the recommendation of the WHO, and the intensity of exercise was far lower than the recommended level, which demonstrates the need to strengthen knowledge, increase self-motivation, and remove barriers.

According to the Knowledge–Attitude–Belief theory, knowledge is the basis of behavior changes. Previous studies found that more than 50% of the population was unaware of the current exercise guidelines [36]. Over half of the respondents did not know how much physical activity was recommended for health benefits. More than one-third of the population underestimated or did not know the increased risk of disease resulting from inactivity [37]. Our previous study explored self-motivation for physical activity and found that children and adolescents participated in physical activity in order to maintain their body size, achieve pleasure at the individual level, and establish social interactions at the interpersonal level, reflecting the importance of developing diverse games to promote physical activity among children and adolescents [38,39]. Sufficient physical activity for children and adolescents encompasses frequency and intensity, as well as the type of activity, and the types of activity should be diverse, including activities for aerobic power, muscle strengthening, balancing, and flexibility [13]. The major concerns about physical activity are a fear of injuries and less study time leading to poor academic performance. However, randomized clinical trials have confirmed that more physical activity does not compromise children’s academic performance [40]. Injuries can be reduced by adequate safety awareness, sufficient warm-up, and suitable exercise environments [41].

Our study showed that 60 min of physical activity four times a week helped to reduce the risk of overweight, with the risk falling by nearly half. A previous study found that physical activity three times a week helped to decrease blood pressure [42]. Additionally, physical activity for at least 4 h a week resulted in weight loss [43]. This suggests that there might be a dose–response relationship between physical activity time and health outcomes and differences in diseases [1].

In our study, we did not find statistical relationships between sex, family economic level, household registration, left-behind situation, and pediatric overweight. This further demonstrates the importance of behavioral interventions in preventing overweight and obesity among children and adolescents [44]. In particular, this study was conducted during the COVID-19 pandemic.

This study has several limitations. First, there is a possible self-report bias within the study. Although the recall duration of physical activity time was limited to the previous 7 days, it is still possible that the participants had memory errors. We used sex- and age-specific BMI as a discriminative indicator for overweight and obesity; however, other indicators such as total lean mass and fat content are also important for childhood overweight and obesity prevention. Future studies should aim to use more objective and diverse indicators. Second, we focused on early, middle, and late puberty. Therefore, the effect of physical activity four times a week on preventing overweight and obesity among children and adolescents pre- and post-puberty is still unknown, and more in-depth studies are needed to further understand the impact of physical activity on childhood overweight and obesity. Third, there is a possible risk of self-selection bias within the study. We excluded participants who did not report their height and weight or whose weight and height were more than 3 times the interquartile range. However, we performed a sensitivity analysis, which included all participants, and it highlighted the robustness of our study findings.

Despite the mentioned limitations, this study is highly significant, as it is one of few to explore how much physical activity is appropriate for a healthy weight in children and adolescents. This is a necessary guide for school-aged children and adolescents with a heavy academic burden and limited leisure time. Therefore, this study can be valuable for further investigations.

## 5. Conclusions

This study aimed to seek an optimal threshold of physical activity for childhood overweight and obesity prevention. In this study, we recommended that children and adolescents engage in physical activity four times a week for 60 min for a healthy weight, which would cut the risk by almost half. These findings can serve as a basis for childhood overweight and obesity guides and public policy development. By exploring the dose–response effect, the study could also provide valuable insights for educators, parents, and health professionals involved in promoting healthy weight in children and adolescents.

## Figures and Tables

**Table 1:** Characteristics of non-overweight and overweight or obese participants (*n* = 842).

	Whole Sample	Non-Overweight	Overweight and Obesity	χ^2^/Mann–Whitney U	*p* *
Sex, *n* (%)				0.929	0.335
male	378(44.9)	280(74.1)	98(25.9)		
female	464(55.1)	357(76.9)	107(23.1)		
Grades, *n* (%)				29.992	<0.001
primary school students	177(21.0)	108(61.0)	69(39.0)		
junior high school students	182(21.6)	135(74.2)	47(25.8)		
senior high school students	483(57.4)	394(81.6)	89(18.4)		
Household registration status, *n* (%)				0.901	0.506
urban household	444(52.7)	330(74.3)	114(25.7)		
rural household	398(47.3)	307(77.1)	91(22.9)		
Left-behind situation, *n* (%)				0.443	0.343
non-left-behind children	781(92.8)	593(75.9)	188(24.1)		
left-behind children	61(7.2)	44(72.1)	17(27.9)		
Family economic level, *n* (%)				0.604	0.739
low	167(19.8)	130(77.8)	37(22.2)		
medium	469(55.7)	351(74.8)	118(25.2)		
high	206(24.5)	156(75.7)	50(24.3)		
Screen exposure duration, median (*IDQ*)	21(11.0)	21(11.0)	20(11.0)	−1.126	0.019

*IDQ*, interquartile range. * Comparisons were made between non-overweight and overweight and obesity groups.

**Table 2:** Impacts of physical activity time on overweight and obesity.

	A*OR*(95% *CI*)	*p*
Sex (male as ref.)		
female	0.89(0.64, 1.23)	0.477
Grades (primary school students as ref.)		
junior high school students	0.53(0.33, 0.82)	0.006
senior high school students	0.34(0.22, 0.51)	<0.001
Household registration status (urban household as ref.)		
rural household	1.02(0.73, 1.44)	0.891
Left-behind situation (non-left-behind children as ref.)		
left-behind children	1.20(0.66, 2.20)	0.547
Family economic level (low as ref.)		
medium	1.04(0.67, 1.62)	0.846
high	0.99(0.59, 1.66)	0.965
Screen exposure duration	0.99(0.97, 1.01)	0.259
Physical activity time (<4 days a week as ref.)		
≥4 days a week	0.56(0.38, 0.83)	0.004

Abbreviations: ref.: reference; A*OR*: adjusted odds ratio.

## Data Availability

All data are presented in this paper.
